# Primary orbital extraskeletal osteosarcoma and review of literature

**DOI:** 10.1186/s12886-020-01690-9

**Published:** 2020-10-22

**Authors:** Jingwen Hui, Yun Zhao, Lei Zhang, Jinyong Lin, Hong Zhao

**Affiliations:** 1 Tianjin Eye Hospital, Tianjin Eye Institute, NO.4 Gansu Road, Heping District, Tianjin, 300020 China; 2 Tianjin Key Lab of Ophthalmology and Visual Science, Tianjin, China; 3 Clinical College of Ophthalmology Tianjin Medical University, Tianjin, China; 4 Nankai University Eye Hospital, Tianjin, China

**Keywords:** Orbit, Osteosarcoma, Extraskeletal osteosarcoma

## Abstract

**Background:**

Extraskeletal osteosarcoma is a malignant tumour composed of an osteoid and/or cartilaginous matrix; it arises in soft tissues without connection to the skeleton, and to our knowledge, this type of tumour is extremely rare.

**Case presentation:**

The present study reports a 57-year-old man with primary orbital extraskeletal osteosarcoma who presented with a history of painful swelling in the left orbit that had occurred for 11 months. Imaging of the orbit showed an atypical, well-defined heterogeneous mass attached to the posterior globe of the left orbit. The patient underwent an anterior orbitotomy and complete excision of the tumour. The mass was originated from neither the globe nor the bony orbital wall but from the soft tissue. Histopathology demonstrated an extraskeletal osteosarcoma. After 13 months of follow-up, there was apparent recurrence of the tumour. The medical history showed no complaints of previous trauma or radiotherapy.

**Conclusions:**

ESOS is a highly malignant tumour. Immunosuppression, trauma and adjuvant radiotherapy are possible predisposing factors in the development of this tumour. Prompt recognition and thorough treatment are essential for preventing orbital lesions and presence of metastasis from other organs.

## Background

Extraskeletal osteosarcoma (ESOS) is an extremely rare tumour that is not usually located in the orbit. It is defined as a malignant mesenchymal neoplasm that produces an osteoid and/or cartilaginous matrix and arises in soft tissues without connection to the skeleton. It is rare and comprises fewer than 5% of all osteosarcomas. Only 4 previous cases of orbital ESOS have been reported. Extraskeletal osteosarcoma primarily affects patients over the age of 50 years and has a poor prognosis. Our patient is the oldest and the fourth case ever detected with primary orbital ESOS without a history of previous radiotherapy or trauma. In this report, we describe the clinical, radiologic, and pathologic records of a rare case of primary orbital extraskeletal osteosarcoma.

## Case presentation

A 57-year-old male presented with painful swelling in the left orbit that had occurred for 11 months. The medical history showed no chronic systemic disease, sinus infection, mucocele, previous trauma, previous eye surgery or irradiation.

**Fig. 1:**
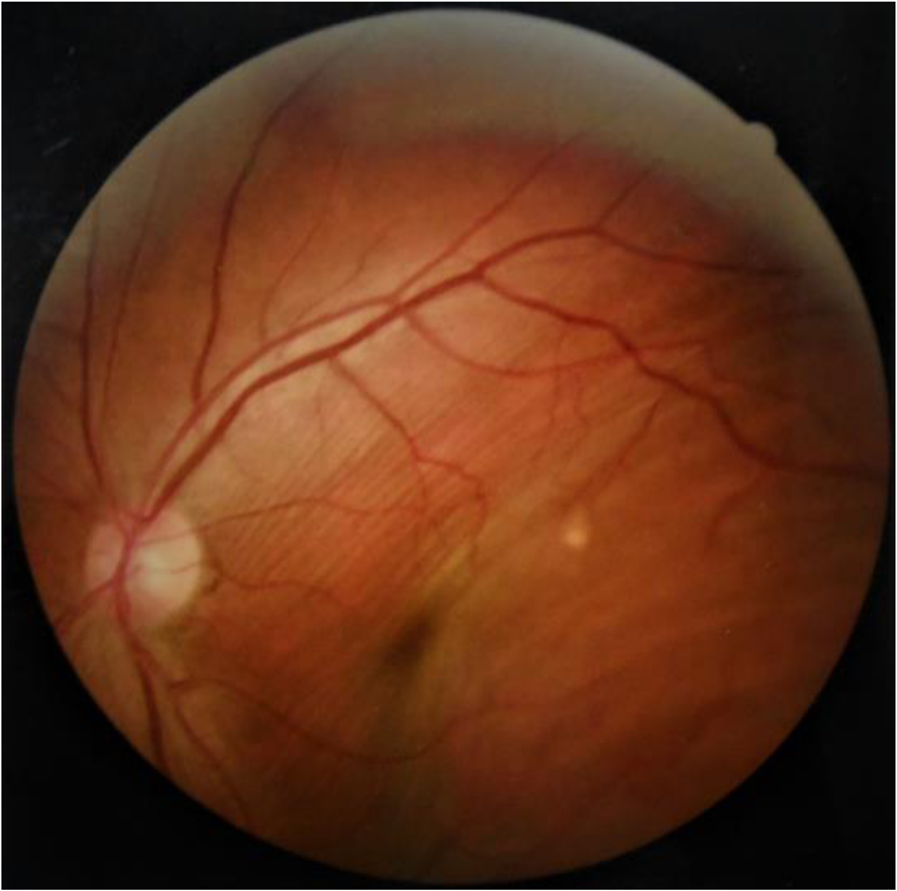
Diffuse choroidal fold was shown in left eye in the fundus photography

We performed a complete ophthalmic examination. Best-corrected visual acuity was 20/25 OD and 20/70 OS. Examination of the left eye revealed a 3 mm proptosis and maximal restriction of extraocular movements in all gazes. Funduscopic examination revealed diffuse choroidal fold in the left eye (Fig. 1). Right eye examination was unremarkable.

**Fig. 2:**
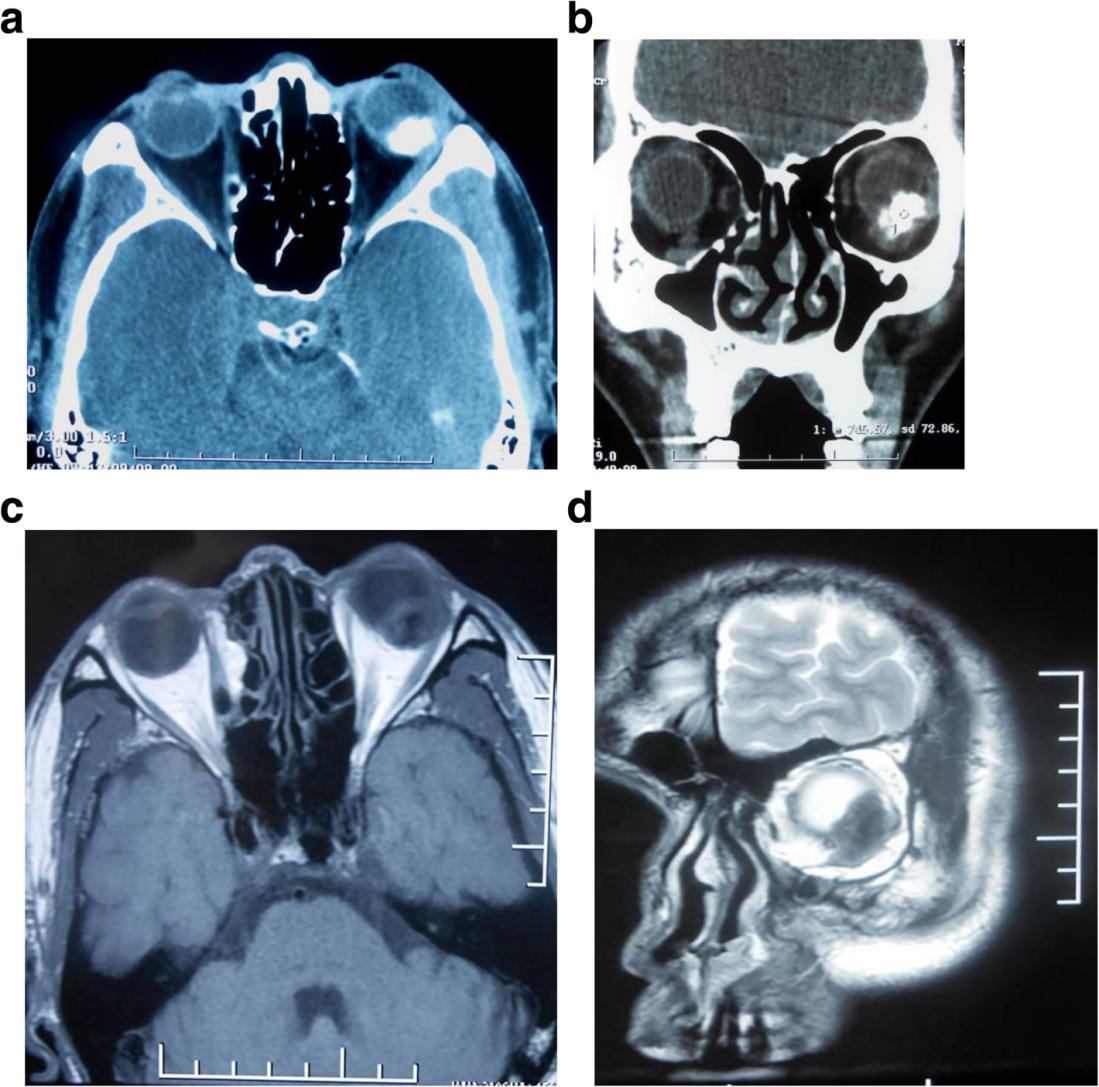
**a**, The transverse computed tomography revealed an atypical, well-circumscribed homogeneous calcified mass attached the posterior globe in the left orbit. **b**, The coronal computed tomography showed a calcified mass neither origined from the globe nor the bony orbital wall. **c**, The transverse T1WI section showed the heterogeneous lesion .The lesion was approximately 17.7 × 14.1 × 24.2 mm in the orbit. **d**, The coronal T2WI section showed the heterogeneous lesion presents hypointense in the left orbit

Computed tomography (CT) of the orbit revealed an atypical, well-circumscribed homogeneous calcified mass attached to the posterior globe of the left orbit (Fig. 2 a-b). Orbital magnetic resonance imaging showed a mixed heterogeneous mass with hypo and hyperintense regions (Fig. 2 c-d). The mass was measured 1.77 × 1.41 × 2.42 cm in size in the left orbit and was located within the muscle cone.

**Fig. 3:**
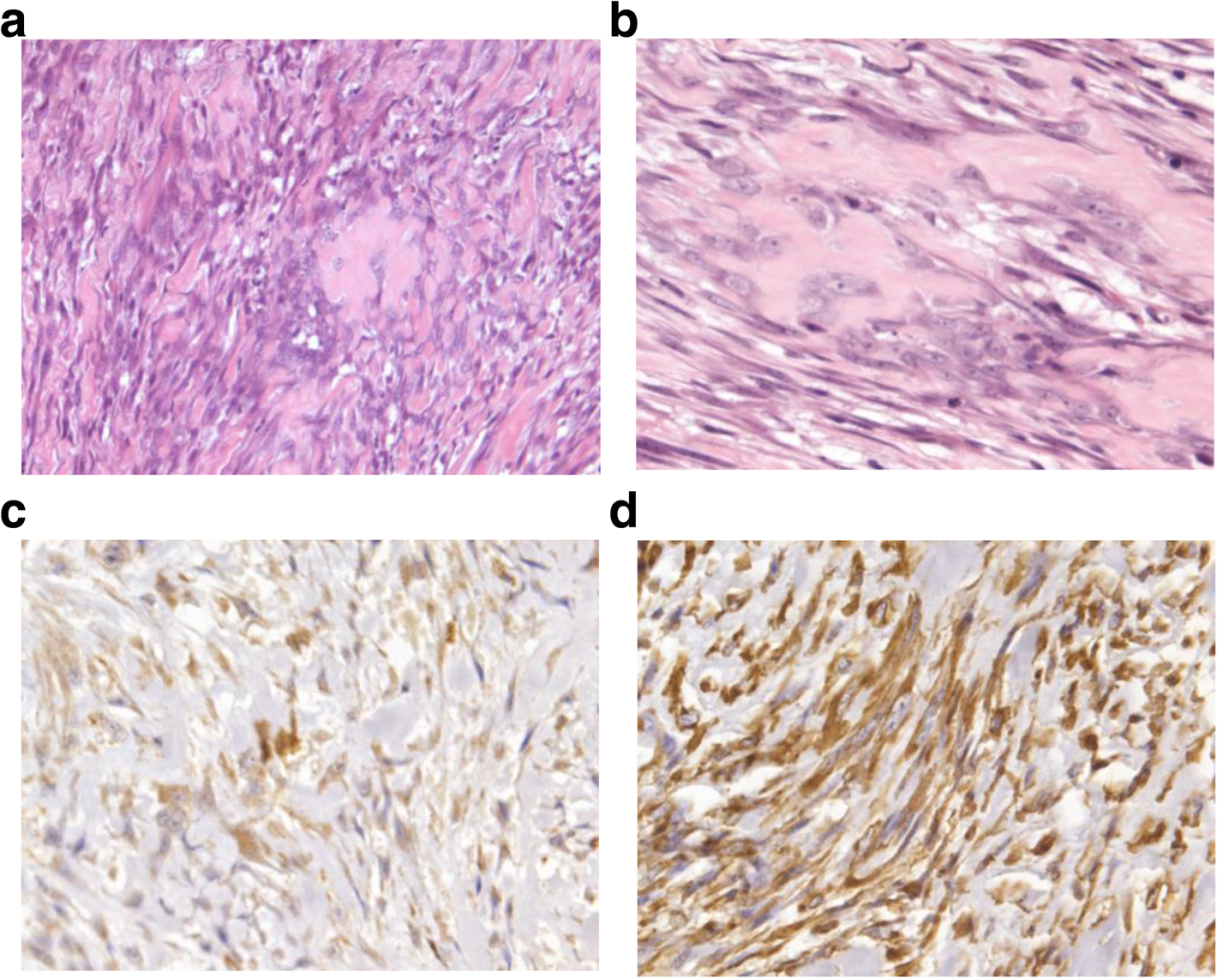
**a** Hematoxylin-eosin staining with low-power view showing bizarre tumor cells and osteoid with atypical mitotic figures. (hematoxylin-eosin, original magnification× 200). **b** High-power view of the cellular area showing malignant spindle cells with abundant neoplastic bone and cartilage formation (hematoxylin-eosin, original magnification× 400). **c-d** Immunohistochemical examination showing positivity of tumor cells to S-100**(c)** and Vimentin **(d)** (original magnification× 400)

The patient underwent an anterior inferior orbitotomy, and complete excision of the mass. Intraoperatively, the tumour was attached to the sclera but not attached to any of the orbital structures. The tumour wraps the inferior oblique, lateral rectus, and part of the inferior rectus muscle. The sclera was intact and the surface was slightly rough. The tumour caused indentation of the globe but did not enter the globe. Gross examination revealed an atypical, well-encapsulated grey-brown bony mass with several small, separate, nodules. The cut surface was grey-white, lobulated and bony hard in consistency. Microscopic examination revealed malignant spindle cells with abundant neoplastic bone and cartilage formation (Fig. 3 a-b). Immunohistochemical examination was positive for vimentin and S-100(Fig. 3 c-d) and negative for desmin, CD99 and EMA. The patient underwent PET-CT examination before surgery and found no distant metastases from this lesion or for occult primary lesions that may have led to a metastatic lesion within the orbit.

**Fig. 4:**
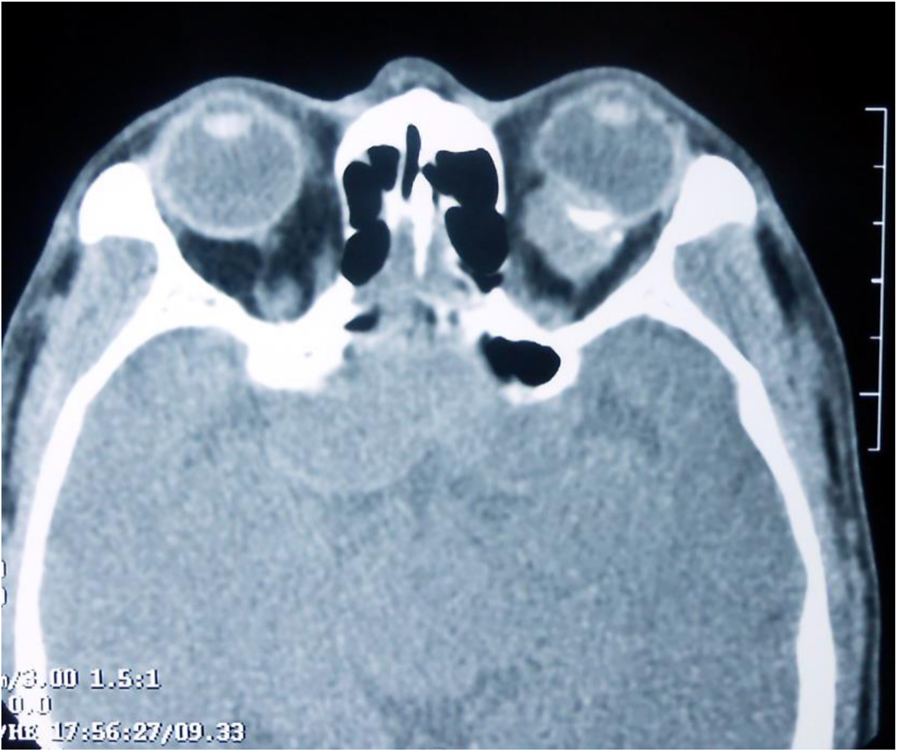
Preoperative CT in the recurrence

The 13-month follow-up included CT scans every 3 months and subsequently every 6 months. The patient encountered recurrence of the tumour. A complete ophthalmic examination was performed. Visual acuity was 20/25 OD and 20/200 OS. Examination of the left eye revealed a 1 mm proptosis and restriction of extraocular movements in all gazes. Funduscopic examination revealed diffuse choroidal fold in the left eye. Right eye examination was unremarkable. The latest orbital CT (Fig. 4) showed an irregular shape of the soft tissue in the muscle cone of the left eyeball. The lesions showed irregular massive calcification, and the tumour was close to the posterior pole, which was compressed and deformed. The boundary between the mass and the medial rectus muscle and the inferior rectus muscle was unclear, which revealed that the muscle was probably attached. The CT value of the soft tissue lesions was 52 ± 7.62 HU, while that of calcification was 319.09 ± 84.31HU. Considering the patient’s medical history, due to the invasive nature of the tumour, the patient then underwent orbital exenteration of the left orbit. The tumour was confirmed after surgery. Pathological examination showed a recurrence of extraskeletal osteosarcoma outside the orbit, and the lesion was extensively invaded by the soft tissue and the posterior sclera.

The pathological diagnosis was recurrent orbital extraskeletal osteosarcoma.

## Discussion and conclusion

ESOS is a rare malignant mesenchymal neoplasm, which located in soft tissue without connection to the skeleton. Only 4 cases of ESOS in the orbital region have been reported in the literature (Table 1) [1–4]. Since it was first described by Wilson [5] in 1941, only a limited range of publications about this rare neoplasm have been published. ESOS is a relatively unusual soft tissue tumour, with an incidence of 1–2% of all soft tissue sarcomas and 4% of all osteosarcomas [6]. Osteosarcoma of the bone occurs mainly around the age of 20, whereas ESOS has been reported in adults with an average age of 52 [7] .The most typical sites of ESOS are the lower extremities, girdle, the thigh muscle, and retroperitoneum [8], while in principle ESOS can arise in any part of the body and rarely occurs in the orbit.

**Table 1:** Cases of extraskeletal osteosarcoma in the orbital region reported in the literature

Ref. No.	Author (yr)	Age (yrs)/Sex	Primary malignancies	Prior history irradiation	Outcome
[1]	Kauffman and Stout (1963)	11/M	Retinoblastoma	Present	NR
[2]	Jacob et al. (1998)	22/M	Absent	Absent	NED
[3]	Fan et al. (2011)	78/M	Basal cell carcinoma	Present	NED
[4]	De Maeyer et al. (2016)	32/M	Absent	Absent	NED

*M* male, *NED* no evidence of disease, *NR* not reported

Research focusing on the clinical behaviour of ESOS is limited. Localized pain, swelling and oedema are the most common presenting symptoms [9]. The duration of symptoms varies from weeks to years and most series report an average duration of 4 ± 6 months [9, 10]. The diagnosis tends to be delayed because symptoms are often absent or vague according to documents [11]. ESOS can be asymptomatic or symptomatic. The presenting symptoms are typically due to tumour enlargement and pain. As with other neoplasms, the aetiology of ESOS is difficult to determine. Radiation and trauma are regarded as major incriminating factors. However, there was no evidence of previous trauma or irradiation in our case.

In our case, the differential diagnosis should be as follows:(1) choroidal osteoma,(2) mesenchymal chondrosarcoma. Choroidal osteoma has not been suggested because the imaging studies showed only the limited lesions of the choroid with the presence of cancellous bone and vascular channels within the choroid, and this type of tumour commonly occurs in healthy females in their 2nd or 3rd decades of life. Without the component of cartilage and accordant pathological examination, the diagnosis of mesenchymal chondrosarcoma was not supported.

The most common genetic changes associated with osteosarcoma are the alterations of the p53 tumour suppressor gene and loss of the retinoblastoma gene RB1on the chromosome [12]. In the present case, alterations of p53 and expression levels of RB1 were not tested. The aetiology of sporadic cases is not clear, appearing to be associated with the cumulative effect of susceptibility loci on other chromosomes controlling the mechanisms of cell proliferation, differentiation, and maturation.

The common metastatic sites of involvement are the lungs, followed by the lymph nodes, kidneys, thyroid, liver, spleen and other viscera [13]. The treatment of ESOS includes radical surgery and polychemotherapy [14]. Treatment of ESOS is primarily surgical with wide excision or radical resection. Radical resection appears to be the best therapeutic option for local control but has no effect on distant metastasis [15], which also does not seem to improve survival in patients treated with wide excision. Adjuvant chemotherapy or preoperative radiation therapy appears to be useful. Radiotherapy can be used in patients with large lesions or marginal excision, while chemotherapy is not commonly used except for palliation. Various chemotherapy protocols have been used in advanced and metastatic disease and the outcome was uniformly poor. Although some cases have been reported to be treated by radiotherapy or systemic chemotherapy, the treatments are not well established.

In summary, ESOS is a highly malignant tumour. If a patient shows the orbital signs of suspicious of ESOS, we recommend that a diagnosis should be promptly performed, and appropriate surgery in terms of excision is a prerequisite. Patients should be carefully monitored with very close follow up and should be warned about symptoms and signs of recurrence after surgery, which can rapidly progress and cause severe visual impairment. Prompt recognition and thorough treatment are essential for preventing orbital lesions and presence of metastasis from other organs.

## Data Availability

All datasets used and/or analysed in the current study are available from the corresponding author upon reasonable request.

## Abbreviations

ESOS: Extraskeletal osteosarcoma
CT: Computed Tomography
T1WI: T1weighted image
T2WI: T2weighted image
EMA: Human Epithelial membrane antigen
HU: Hounsfield unit
